# Calprotectin in the risk stratification of patients with acute dyspnoea in the emergency department

**DOI:** 10.1038/s41598-025-07741-9

**Published:** 2025-06-27

**Authors:** Martin Wollmer, Torgny Wessman, Anders Larsson, Anna C. Nilsson, Olle Melander, Toralph Ruge

**Affiliations:** 1 Department of Emergency and Internal Medicine, Skåne University Hospital, Ruth Lundskogs gata 5, 205 02 Malmö, Sweden; 2 Department of Clinical Sciences Malmö, Lund University, Lund, Sweden; 3 Department of Medical Sciences, Uppsala University, Uppsala, Sweden; 4 Department of Infectious Disease Medicine, Skåne University Hospital, Malmö, Sweden; 5 Infectious Disease Research Unit, Department of Translational Medicine, Lund University, Malmö, Sweden

**Keywords:** Emergency service, hospital, Leukocyte L1 antigen complex, Biomarkers, Dyspnea, Communicable diseases, Mortality, Prognostic markers, Cardiovascular diseases, Infectious diseases

## Abstract

Acute dyspnoea is one of the most common presenting symptoms in the emergency department (ED) and has a variety of underlying causes. Calprotectin is a neutrophil activation marker associated with adverse outcomes in acute cardiovascular and infectious diseases. However, the usefulness of calprotectin in the risk stratification of patients with acute dyspnoea is unknown. The objectives were to, in unselected patients presenting to the ED with acute dyspnoea, investigate the association between (1) calprotectin and 90-day mortality, (2) calprotectin and 90-mortality in subgroups of patients with cardiovascular disease or pneumonia, and (3) calprotectin and illness severity. Single-centre observational cohort study from a university hospital in southern Sweden. A total of 1186 patients from the original Acute Dyspnoea Study, were included. Patients were followed for discharge diagnosis and mortality. Calprotectin concentration was measured in plasma samples collected at the ED. Mean age was 72 years and 56% were women. During follow-up, 143 patients died. In multivariate Cox regression for 90-day mortality, calprotectin in the highest quartile (> 0.96 mg/L) compared to the lowest quartile (< 0.27 mg/L) was associated with a hazard ratio of 2.71 (95% confidence interval 1.39–5.26, *p* < 0.01). The association with mortality remained significant in the subgroup of patients with acute cardiovascular disease (*N* = 205, *p* < 0.01). There was no statistically significant difference in median calprotectin values between survivors and non-survivors with pneumonia (1.62 vs. 1.31, *p* = 0.155). Multivariate linear regression showed a strong positive correlation between calprotectin and illness severity (respiratory rate ≥ 29 or oxygen saturation ≤ 90%, *p* < 0.001). In conclusion, calprotectin was associated with 90-day mortality and correlated strongly with illness severity. This indicates that measurement of calprotectin at admission could improve clinical risk stratification of the acute dyspnoeic ED patient.

**Clinical trial number**: Not applicable.

**Supplementary Information:**

The online version contains supplementary material available at 10.1038/s41598-025-07741-9.

## Introduction

Acute dyspnoea is one of the most common presenting symptoms in the emergency department (ED) and is challenging for the emergency physician. The subjective experience of dyspnoea and the variety of underlying causes, particularly in elderly patients with multiple comorbidities, often makes the diagnostic assessment difficult. Investigations beyond history and clinical examination, such as laboratory and radiological examinations, are frequently required. This means that risk stratification must be performed simultaneously with diagnostic assessment. A common aid to improve risk stratification in the emergency department is biomarkers^1,2^.

Calprotectin, a heterodimer of S100A8 and S100A9, is primarily found in the cytosol of neutrophils. It is released upon neutrophil activation and acts as a mediator in the immune response^3^. Calprotectin has been hypothesised to be an early marker of inflammation and studies have shown that circulating calprotectin increases as early as 1.5 h, peaks at 5 h and remains elevated at 24 h after exposure to endotoxin or bacteria^4,5^. C-reactive protein (CRP) is an acute-phase protein and the most commonly used biomarker in Swedish EDs to detect inflammation and infection. It is synthesised by the liver in response to inflammatory stimuli and circulating levels start rising after about six hours and peaks around 48 h^6^.

Calprotectin is routinely analysed in faeces in the evaluation of inflammatory bowel disease and circulating calprotectin is associated with disease activity in chronic inflammatory rheumatic diseases^7,8^. More recently, studies have shown an association between increased levels of circulating calprotectin and adverse outcomes in acute cardiovascular and infectious diseases^9–12^. However, the prognostic value of calprotectin in acute dyspnoea is so far unknown. The aim of this study is to investigate the association between circulating calprotectin and mortality as well as illness severity in patients presenting to the ED with acute dyspnoea.

## Methods

### Study population

The study was performed by analysing a sub cohort of the Acute DYspnoea Study (ADYS)^13^. ADYS was a prospective observational cohort study conducted at the ED of Skåne University Hospital in Malmö, Sweden. Inclusion criteria were age 18 years or older and acute dyspnoea as the presenting symptom. Patients transferred directly from the resuscitation room to the intensive care unit (ICU) and patients with altered level of consciousness were excluded, as they were not able to give informed consent. Inclusion was made by research nurses daytime on working days between 6 March 2013 and 15 January 2019 and a total of 1745 unique patients were included.

### Clinical parameters

Patients were triaged according to Medical Emergency Triage and Treatment System (METTS) into one of four priority levels of increasing urgency: green, yellow, orange or red^14^. The priority level is assigned by combining two different algorithms, one including vital signs and one including emergency symptoms and signs related to the presenting symptom. Due to a low number of patients in the green priority level (*N* = 61), patients in the green and yellow priority level were merged into a new variable for analysis. Height and weight were recorded from the electronic medical records if recent information was available or else obtained during interview of the patient in the ED by a research nurse. The patients were divided into four categories according to body mass index (BMI) (< 18.5, 18.5–24.9, 25.0–29.9 and ≥ 30). Respiratory rate (RR) was calculated manually by an ED nurse. Oxygen saturation (SpO_2_) was measured with a fully automated oscillometric device (CARESCAPE Monitor B850 or B650, General Electric Healthcare, Danderyd, Sverige). Level of consciousness was determined according to the Reaction Level Scale^15^. Information on date of death, cardiovascular comorbidity and final discharge diagnosis was obtained from nationwide registries at the Swedish National Board of Health and Welfare. The cardiovascular comorbidity diagnoses were obtained as groups of International Classification of Diseases, 10th revision (ICD-10) codes including all diagnoses from inpatient and outpatient hospital care from 2012 up until the date of ED visit (see Supplementary Table S1 online). Diseases considered as cardiovascular comorbidity were atrial fibrillation, cardiac valve disease, cerebrovascular disease, heart failure, hypertension, ischemic heart disease and peripheral vascular disease. The final discharge diagnosis was obtained as a specific ICD-10 code. Cardiovascular disease (CVD) as final discharge diagnosis was defined in the same way as cardiovascular comorbidity and pneumonia was defined as those ICD-10 codes considered likely to represent bacterial pneumonia (see Supplementary Table S2). Patients who had an inpatient discharge diagnosis linked to the ED visit were regarded as hospitalised. Blood samples were drawn within one hour of presentation to the ED and then frozen within two hours and stored at -80 °C until analysis.

### Primary and secondary outcomes

The primary outcome was association between calprotectin and 90-day all-cause mortality. Secondary outcomes were association between: (i) calprotectin and 90-day all-cause mortality in hospitalised patients discharged with a final diagnosis of CVD or pneumonia, and (ii) calprotectin and illness severity at presentation to the ED. Illness severity was defined as elevated RR and decreased SpO_2_.

### Biochemical analyses

Calprotectin was analysed in EDTA plasma samples with particle-enhanced turbidimetric assay (PETIA) on a Mindray BS380 chemistry analyser (Mindray, Shenzhen, China) using calprotectin reagents from Gentian AS (Moss, Norway).

CRP was analysed according to local accredited routine with PETIA on Roche Cobas c501, c502 or c701 analysers (Roche Diagnostics, Solna, Sweden).

### Statistical analysis

Continuous variables with normal distribution are presented as mean value with standard deviation (SD) and variables with skewed distribution are presented as median value with inter-quartile range (IQR). T-test or Mann-Whitney U test were used to test for differences between groups, according to distribution. Categorical variables are presented as frequency with proportion. Chi-square test or, if the expected cell count was less than 5, Fischer’s exact test, was used to test for differences between groups. Associations between calprotectin and baseline characteristics or other biomarkers were analysed using univariate linear regression. Hazard ratios (HR) and 95% confidence intervals (CI) for 90-day mortality were estimated using Cox regression. BMI 18.5–24.9 and the merged variable of METTS green and yellow were used as reference categories for these variables. Association between calprotectin and illness severity was analysed using multiple linear regression. Calprotectin was used log-transformed or stratified into quartiles (Q) in regression analyses. CRP was used log-transformed in regression analyses. All analyses were two-tailed and a p-value < 0.05 was considered statistically significant. All statistical analyses were computed with IBM SPSS Statistics version 28.0.

## Results

### Study population

**Fig. 1:**
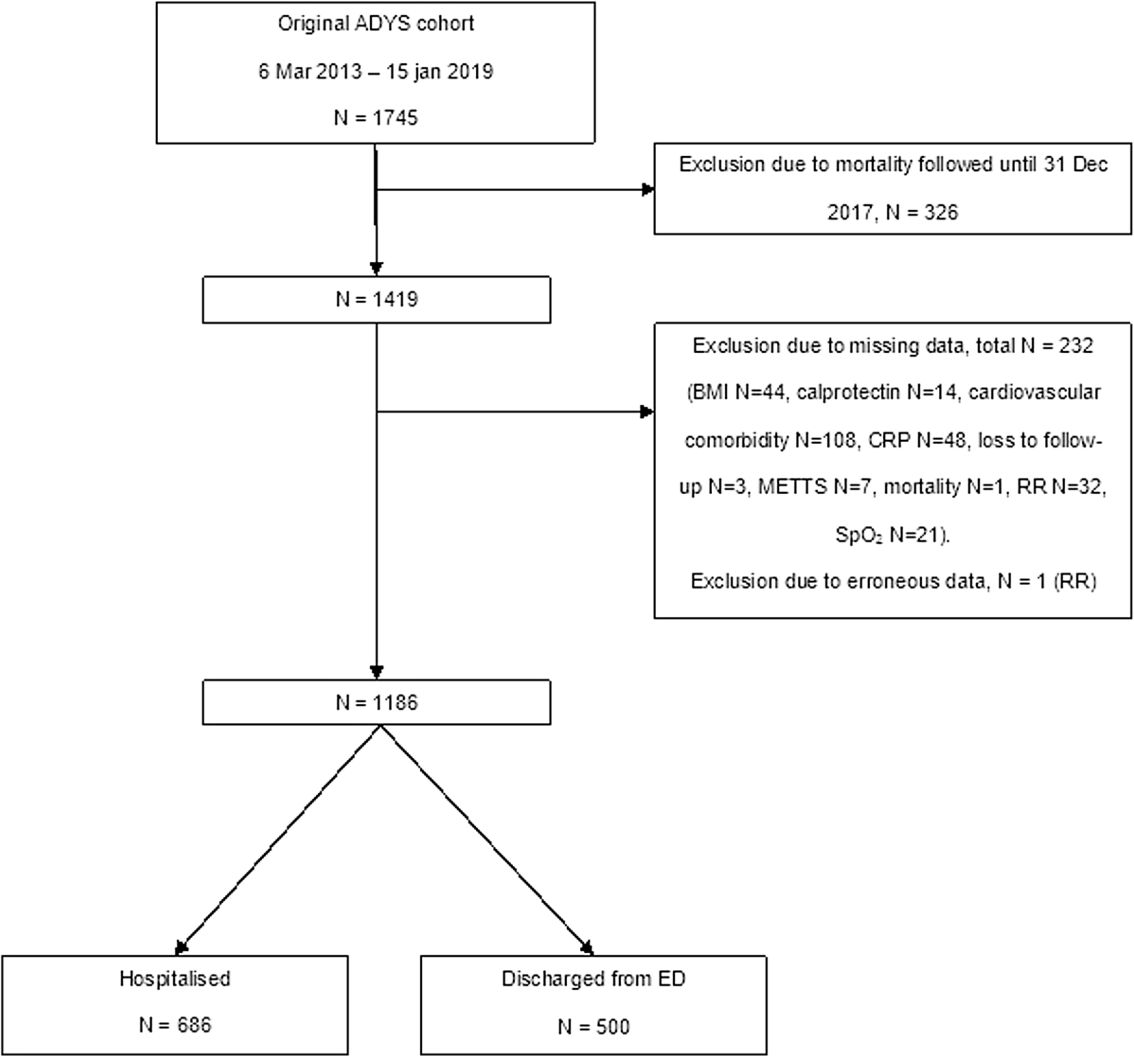
Flowchart of inclusion and exclusion. *ADYS* Acute DYspnea Study, *BMI* Body mass index, *CRP* C-reactive protein, *ED* Emergency department, *METTS* Medical Emergency Triage and Treatment System, *RR* Respiratory rate, *SpO*_*2*_ Oxygen saturation.

Of the 1745 patients in the original cohort, 326 patients were excluded due to mortality being followed until 31 December 2017. Among the remaining 1419 patients, 232 were excluded due to missing data and one was excluded due to erroneous data (respiratory rate 94 breaths/min), leaving a total of 1186 patients eligible for analysis (Fig. 1). A total of 143 (12%) patients were dead at 90 days.

Table 1 shows the baseline characteristics of the 1186 included patients. Correlation between calprotectin and baseline characteristics was analysed with univariate linear regression and showed statistically significant results for age, METTS orange, METTS red, CRP, cardiovascular comorbidity, SpO_2_ and RR (Table 2).

**Table 1:** Baseline characteristics.

Variable	All	Pneumonia	CVD	*p*-value
	*N* = 1186	*N* = 71	*N* = 205	
Female	660 (56%)	35 (49%)	97 (47%)	0.774
Age (years)	72 (± 16)	79 (± 14)	81 (± 11)	0.153
BMI (kg/m^2^)				0.002
< 18.5	64 (5%)	8 (11%)	6 (3%)	
18.5–24.9	448 (38%)	34 (48%)	68 (33%)	
25.0–29.9	385 (33%)	16 (23%)	77 (38%)	
≥ 30	289 (24%)	13 (18%)	54 (26%)	
RR (breaths/min)	24 (± 7)	27 (± 7)	25 (± 7)	0.104
SpO_2_ (%)	95 (90–98)	92 (87–95)	94 (90–97)	0.005
CRP (mg/L)	10 (3–34)	115 (48–213)	10 (4–23)	< 0.001
Calprotectin (mg/L)	0.49 (0.26–0.96)	1.49 (0.90–2.43)	0.41 (0.24–0.67)	< 0.001
METTS				0.162
Green & yellow	655 (55%)	23 (32%)	93 (45%)	
Orange	382 (32%)	35 (49%)	82 (40%)	
Red	149 (13%)	13 (18%)	30 (15%)	
90-day mortality	143 (12%)	15 (21%)	41 (20%)	0.839
Hospitalisation	686 (58%)			
Cardiovascular comorbidity	913 (77%)	59 (83%)	205 (100%)	< 0.001

Baseline characteristics of all patients and hospitalised patients discharged with final diagnosis pneumonia or CVD. Statistical measures are shown as Mean (± SD), Median (IQR) or Number (%). P-values refer to differences between the subgroups of patients with pneumonia and CVD.

*BMI* Body mass index, *CRP* C-reactive protein, *METTS* Medical Emergency Triage and Treatment System, *RR* Respiratory rate, *SpO*_*2*_ Oxygen saturation.

**Table 2:** Correlations between calprotectin and baseline characteristics (*N* = 1186).

Variable	Calprotectin		
	Unstandardised coefficient	95% confidence interval	p-value
Sex	0.05	-0.07 to 0.17	0.383
Age (years)	0.01	0.006 to 0.01	< 0.001
BMI (kg/m^2^)			
< 18.5	0.25	-0.02 to 0.53	0.07
18.5–24.9	Reference	Reference	Reference
25-29.9	-0.07	-0.22 to 0.07	0.321
≥ 30	0.07	-0.08 to 0.23	0.35
METTS			
Green & yellow	Reference	Reference	Reference
Orange	0.36	0.23 to 0.49	< 0.001
Red	0.76	0.58 to 0.94	< 0.001
CRP (mg/L)	0.43	0.40 to 0.46	< 0.001
Cardiovascular comorbidity	0.26	0.12 to 0.40	< 0.001
SpO_2_ (%)			
Q1: ≤90	0.94	0.76 to 1.12	< 0.001
Q2: 91–95	0.50	0.33 to 0.68	< 0.001
Q3: 96–98	0.29	0.12 to 0.47	0.001
Q4: ≥99	Reference	Reference	Reference
RR (breaths/min)			
Q1: ≤20	Reference	Reference	Reference
Q2: 21–24	0.28	0.13 to 0.43	< 0.001
Q3: 25–28	0.38	0.21 to 0.54	< 0.001
Q4: ≥29	0.67	0.51 to 0.83	< 0.001

Correlations were analysed with univariate linear regression.

*BMI* Body mass index, *CRP* C-reactive protein, *METTS* Medical Emergency Triage and Treatment System, *Q* Quartile, *RR* Respiratory rate, *SpO*_*2*_ Oxygen saturation.

### Primary outcome

Table 3 shows the results of Cox regression analysis of 90-day mortality and calprotectin in quartiles (Q1: <0.27 mg/L, Q2: 0.27–0.48 mg/L, Q3: 0.49–0.96 mg/L, Q4: >0.96 mg/L), with the lowest quartile as reference category. The number of deaths were 17 in Q1, 24 in Q2, 32 in Q3 and 70 in Q4. The final model adjusted for age, sex, BMI, METTS, CRP and cardiovascular comorbidity, resulted in a HR of 2.71 (95% CI 1.39–5.26, *p* = 0.003) for Q4 vs. Q1. Other covariates showing a statistically significant association in the final model were sex (*p* < 0.05), age (*p* < 0.001), BMI < 18.5 (*p* < 0.05) and METTS red (*p* < 0.05).

**Table 3:** Cox regression of 90-day mortality (*N* = 1186).

	Model A	Model B	Model C	Model D
Quartile 1	Reference	Reference	Reference	Reference
Quartile 2				
HR	1.52	1.30	1.15	1.16
95% CI	0.82–2.83	0.70–2.42	0.61–2.17	0.62–2.19
p-value	0.188	0.408	0.660	0.644
Quartile 3				
HR	1.98	1.57	1.26	1.26
95% CI	1.10–3.56	0.87–2.83	0.67–2.36	0.67–2.36
p-value	0.023	0.133	0.471	0.469
Quartile 4				
HR	4.87	4.02	2.71	2.71
95% CI	2.86–8.27	2.36–6.83	1.39–5.26	1.39–5.26
p-value	< 0.001	< 0.001	0.003	0.003

Cox regression of 90-day mortality across quartiles of calprotectin, with the lowest quartile as reference category. Results are presented as hazard ratios with 95% confidence intervals.

*Model A*: Calprotectin; *Model B*: Model A, sex and age; *Model C*: Model B, BMI, METTS and CRP; *Model D*: Model C and cardiovascular comorbidity.

### Secondary outcomes

Of the 1186 included patients, 686 were hospitalised after admission to the ED. Among the hospitalised patients two subgroups were identified based on final discharge diagnosis, i.e. CVD (*N* = 205) and pneumonia (*N* = 71). Table 4 shows the distribution of the different CVD diagnoses. Forty-one (20%) patients in the CVD group died within 90 days, of which 12 had calprotectin > 0.96 mg/L. Fifteen (21%) patients in the pneumonia group died within 90 days, of which 10 had calprotectin > 0.96 mg/L. Baseline characteristics are shown in Table 1. There were statistically significant differences between the two groups with respect to CRP (*p* < 0.001), calprotectin (*p* < 0.001), cardiovascular comorbidity (*p* < 0.001), BMI (*p* < 0.01) and SpO_2_ (*p* < 0.01). In the pneumonia subgroup, there was no significant difference in the median values of calprotectin between survivors and non-survivors at 90 days (Median (IQR) 1.62 (0.91–2.96) vs. 1.31 (0.63–1.55), *p* = 0.155).

**Table 4:** Distribution of cardiovascular diseases as final discharge diagnosis.

Discharge diagnosis	*N* (%)	Calprotectin (mg/L)
Heart failure	160 (78)	0.40 (0.04–4.18)
Ischemic heart disease	19 (9)	0.44 (0.04–4.54)
Valvular heart disease	3 (2)	0.42 (0.16–0.64)
Atrial fibrillation/flutter	22 (11)	0.35 (0.10–15.45)
Ischemic stroke	1 (1)	0.86 (0.86)

Final discharge diagnosis in patients hospitalised due to cardiovascular disease (*N* = 205). Results are shown as Number (%) and Median (Min – Max).

Table 5 shows the results of Cox regression analysis of 90-day mortality and log-transformed calprotectin in the CVD subgroup. There was a statistically significant association between calprotectin and mortality. None of the other covariates showed any statistically significant association in the final model.

**Table 5:** Cox regression CVD subgroup.

	HR	95% CI	*p*-value
Model A	1.94	1.45–2.59	< 0.001
Model B	1.90	1.42–2.54	< 0.001
Model C	1.82	1.17–2.83	0.008

Cox regression of 90-day mortality and log-transformed calprotectin in hospitalised patients discharged with final diagnosis cardiovascular disease (*N* = 205). Results are presented as hazard ratios with 95% confidence intervals.

*Model A*: Calprotectin; *Model B*: Model A, sex and age; *Model C*: Model B, BMI, METTS and CRP.

Table 6 shows the results of multiple linear regression analysis of log-transformed calprotectin and illness severity, adjusted for age and sex. Calprotectin correlated positively with increasing respiratory rate (Q2 vs. Q1, *p* < 0.05; Q3 vs. Q1, *p* < 0.05; Q4 vs. Q1, *p* < 0.001) and decreasing SpO_2_ (Q3 vs. Q4, *p* < 0.01; Q2 vs. Q4, *p* < 0.001; Q1 vs. Q4, *p* < 0.001).

**Table 6:** Correlation between calprotectin and illness severity (*N* = 1186).

Variable	Calprotectin		
	Unstandardised coefficient	95% confidence interval	p-value
Sex	0.04	-0.07–0.15	0.494
Age (years)	0.003	-0.001–0.007	0.115
SpO_2_ (%)			
Q1: ≤90	0.75	0.56–0.95	< 0.001
Q2: 91–95	0.44	0.27–0.62	< 0.001
Q3: 96–98	0.26	0.08–0.44	0.005
Q4: ≥99	Reference	Reference	Reference
RR (breaths/min)			
Q1: ≤20	Reference	Reference	Reference
Q2: 21–24	0.19	0.05–0.34	0.01
Q3: 25–28	0.20	0.03–0.37	0.019
Q4: ≥29	0.40	0.23–0.57	< 0.001

Multivariate linear regression with log-transformed calprotectin, age, sex and clinical markers of illness severity (RR and SpO_2_).

*Q* quartile, *RR* Respiratory rate, *SpO*_*2*_ Oxygen saturation.

## Discussion

In this study of unselected patients admitted to the ED with acute dyspnoea we show that increased plasma calprotectin is independently associated with 90-day mortality. Calprotectin in the highest quartile is associated with 2.7 times increased risk of death compared to calprotectin in the lowest quartile. The association remained statistically significant in subgroup analysis of patients hospitalised due to CVD. We also found a significant positive correlation between calprotectin and illness severity at presentation to the ED.

To our knowledge we are the first to report an association between calprotectin and mortality in unselected ED patients with acute dyspnoea. Shaub et al. analysed the predictive ability of serum calprotectin for all-cause mortality in ED patients presenting with acute chest pain^16^. In a Cox regression model adjusted for clinical risk factors and high-sensitivity cardiac troponin T, calprotectin in the highest tertile was an independent predictor. After three biomarkers of plaque instability were added to the model, including CRP, only CRP remained an independent predictor. These conflicting results could be explained by differences in inclusion criteria and duration of follow-up. Larsson et al. reported significantly higher calprotectin and an AUC of 0.64 for unselected patients who died within 30 days of admission to a general ICU, but calprotectin was not an independent predictor in multivariate analysis^17^. Calprotectin is known to be associated with mortality in more selected cohorts of patients, i.e. ST-elevation myocardial infarction (STEMI), acute ischemic stroke, COVID-19 and sepsis treated in the ICU^9–12^. Our results suggest that calprotectin may be a more general marker of mortality than previously known.

The results in the subgroup analysis show that increasing calprotectin is associated with mortality in patients hospitalised due to CVD. Increased levels of inflammatory biomarkers have previously been shown to be predictive of adverse outcomes in acute cardiovascular diseases. CRP has been shown to be an independent predictor of adverse outcomes at 90 days in patients with unstable angina, 30-day mortality in patients with acute heart failure and 90-day mortality in patients with acute ischemic stroke^18–20^. Furthermore, the neutrophil activation marker myeloperoxidase has been shown to be an independent predictor of major adverse cardiac events at 30 days in patients presenting to the ED with suspected acute coronary syndrome^21^. Neutrophils are known to be a part of the pathophysiologic process in both the acute and chronic state of several cardiovascular diseases^22^. As calprotectin is a marker of neutrophil activation it is not surprising to find an association with mortality in the patients experiencing acute dyspnoea due to CVD. This finding is also consistent with previous observations in STEMI and acute ischemic stroke, despite our study population being more heterogenous^9,12^. However, in contrast to previous studies, we did not find any statistically significant association between CRP and mortality in multivariate analysis. This could be explained by the differences in study populations. Another possible explanation is the different dynamics of CRP and calprotectin. CRP peaks around 48 h after the inflammatory stimulus while calprotectin peaks at 5 h^4–6^. Theoretically, calprotectin could thus be a more suitable marker for risk stratification in the ED.

The research on calprotectin and prognosis of pneumonia is conflicting. One study in patients hospitalised with community-acquired pneumonia (CAP) found serum S100A9 to be associated with inhospital mortality in a logistic regression model adjusted for age and sex^23^. Another study in hospitalised patients with CAP did not find any significant association between calprotectin and a composite endpoint of ICU admission and 30-day mortality^24^. We did not find any statistically significant difference in median calprotectin values between survivors and non-survivors in the pneumonia subgroup, even though 10/15 patients who died had calprotectin > 0.96 mg/L. Unfortunately, the small sample size (*N* = 71) makes the analysis under-powered and it should be viewed as exploratory. However, as the previous research is limited and conflicting, it highlights the need for further research.

Our results show a strong positive correlation between calprotectin and illness severity at presentation to the ED. Recent studies have shown associations between elevated calprotectin and severity of COVID-19, however our cohort was collected before the COVID-19 pandemic^25–27^. Neutrophils are an important part of an effective and regulated immune response against infection^28^. Both reduced neutrophil activity in elderly patients with CAP and excessive neutrophil activity in sepsis-associated acute respiratory distress syndrome (ARDS) is associated with worse outcomes^28,29^. It is an intriguing question if calprotectin, as a marker of neutrophil activity, can differentiate between a regulated immune response against infection and the dysregulated response associated with respiratory dysfunction in sepsis.

### Clinical implications

The purpose of risk stratification at ED admission is to identify patients with the highest risk of adverse outcomes and prioritise them for physician assessment. Our results suggest that calprotectin > 0.96 mg/L can identify a group at high risk of 90-day mortality in unselected patients with acute dyspnoea, independently of a triage system (METTS), CRP and cardiovascular comorbidities. However, further research is needed before clinical implementation. Calprotectin should be studied in a larger sample of unselected patients, i.e. regardless of presenting symptom. Furthermore, an appropriate cut-off must be established before the clinical utility of calprotectin can be determined. Of particular interest would be to investigate the ability of calprotectin to identify high-risk patients that were assigned a low priority by the triage system. Furthermore, calprotectin may represent a valuable biomarker for risk stratification of patients with pneumonia and CVD, particularly in informing disposition decisions following clinical evaluation and preliminary diagnosis. For instance, elevated calprotectin could support early initiation of antibiotic therapy. Our analysis found an association between elevated calprotectin and mortality in patients with CVD, indicating that these patients may be at higher risk. Further studies in larger sample sizes, including patients of all acuity levels, are required to explore the potential value of calprotectin in risk stratification of patients with pneumonia and CVD.

### Limitations and strengths

This study has several limitations. First, it is an observational study and we cannot draw conclusions about causality. Second, the study was performed at a single centre and included patients based on one symptom so we cannot generalise to other settings or patient populations. Third, the discharge diagnoses used to define the subgroups were made by the treating physician and there is a risk of misclassification. To minimise this risk, we only included patients treated in hospital as the diagnosis was then made at the end of hospitalisation. The subgroup of patients with pneumonia was also relatively small and the results should be interpreted with caution. In contrast to calprotectin, the value of CRP was available to the ED physician which may have influenced the discharge diagnosis. However, as measurement of CRP is a routine part of the assessment of suspected infection in our ED, it would have been unethical to withhold it from the treating physician. Last, the regression analysis of illness severity was not adjusted for oxygen supplementation which could have influenced the results. Strengths of the study include a large sample size that is well characterised, unselected inclusion and a nationwide coverage of comorbidity diagnoses.

## Conclusion

Measurement of calprotectin at admission could improve clinical risk stratification of the acute dyspnoeic ED patient.

## Electronic supplementary material

Below is the link to the electronic supplementary material.

Supplementary Material 1

## Data Availability

The dataset analysed during the current study is available from the corresponding author upon reasonable request.

## Abbreviations

ADYS: The Acute Dyspnoea Study
ARDS: Acute respiratory distress syndrome
BMI: Body mass index
CAP: Community-associated pneumonia
CI: Confidence interval
CRP: C-reactive protein
CVD: Cardiovascular disease
ED: Emergency department
HR: Hazard ratio
ICD-10: International Classification of Diseases, 10th revision
ICU: Intensive care unit
IQR: Inter-quartile range
METTS: Medical Emergency Triage and Treatment System
PETIA: Particle-enhanced turbidimetric assay
Q: Quartile
RR: Respiratory rate
SD: Standard deviation
SpO_2_: Oxygen saturation
STEMI: ST-elevation myocardial infarction
