# Supplementary material for: Calprotectin in the risk stratification of patients with acute dyspnoea in the emergency department
Source: Sci Rep. 2025 Jun 27;15:20336. doi: 10.1038/s41598-025-07741-9 (PMC12205072; doi:10.1038/s41598-025-07741-9)
Supplement: Supplementary file 1 — Supplementary Material 1 [file 41598_2025_7741_MOESM1_ESM.docx]

Supplementary Table S1. Cardiovascular comorbidity diagnoses

| **Atrial fibrillation** |
| --- |
| I48 |
| **Cardiac valve disease** |
| I05 |
| I06 |
| I07 |
| I08 |
| I091 |
| I098 |
| I34 |
| I35 |
| I36 |
| I37 |
| I38 |
| I390 |
| I391 |
| I392 |
| I393 |
| I394 |
| Q22 |
| Q23 |
| Z952 |
| Z953 |
| Z954 |
| **Cerebrovascular disease** |
| G45 |
| G46 |
| I60 |
| I61 |
| I62 |
| I63 |
| I64 |
| I67 |
| I69 |
| **Heart failure** |
| I110 |
| I130 |
| I132 |
| I27 |
| I280 |
| I42 |
| I43 |
| I50 |
| I515 |
| I517 |
| I528 |
| Z941 |
| Z943 |
| **Hypertension** |
| I10 |
| I11 |
| I12 |
| I13 |
| I15 |
| **Ischemic heart disease** |
| I20 |
| I21 |
| I22 |
| I24 |
| I25 |
| Z951 |
| Z955 |
| **Peripheral vascular disease** |
| I702 |
| I73 |
| I792 |
| I798 |
| Excluded ICD codes: I731, I738 |

Grouping of ICD-10 codes used for extraction of cardiovascular comorbidity from the national register

Supplementary Table S2. Discharge diagnoses

| **Cardiovascular disease** |
| --- |
| I110 |
| I200 |
| I209 |
| I214 |
| I214a |
| I219 |
| I350 |
| I352 |
| I480 |
| I482 |
| I489 |
| I500 |
| I501 |
| I509 |
| I634 |
| **Bacterial pneumonia** |
| J149 |
| J157 |
| J158 |
| J159 |
| J189 |

ICD-10 codes considered cardiovascular disease or bacterial pneumonia as final discharge diagnosis
